# Melatonin Administration Accelerates Puberty Onset in Mice by Promoting FSH Synthesis

**DOI:** 10.3390/molecules26051474

**Published:** 2021-03-09

**Authors:** Chan Yang, Zaohong Ran, Guoshi Liu, Rong Hou, Changjiu He, Qinghua Liu, Yingjun Chen, Yuliang Liu, Xiaodong Wang, Chuqi Ling, Fang Fang, Xiang Li

**Affiliations:** 1 Key Laboratory of Agricultural Animal Genetics, Breeding and Reproduction of Ministry of Education, National Center for International Research, Department of Hubei Province Engineering Research Center in Buffalo Breeding and Products, College of Animal Science and Technology, Huazhong Agricultural University, Wuhan 430070, China; YangChan@webmail.hzau.edu.cn (C.Y.); RanZaohong@webmail.hzau.edu.cn (Z.R.); chungjoe@mail.hzau.edu.cn (C.H.); 2017302110041@webmail.hzau.edu.cn (Q.L.); cyjmail.hzau.edu.cn@webmail.hzau.edu.cn (Y.C.); 2015302200616@webmail.hzau.edu.cn (X.W.); Chuqiling@outlook.com (C.L.); fangfang@mail.hzau.edu.cn (F.F.); 2 College of Animal Science and Technology, China Agricultural University, Beijing 100083, China; gshliu@cau.edu.cn; 3 Chengdu Research Base of Giant Panda Breeding, Chengdu 610081, China; hourong2000@panda.org.cn (R.H.); liuyuliang@panda.org.cn (Y.L.)

**Keywords:** melatonin, puberty onset, reproductive hormones, pituitary, ovary

## Abstract

Although melatonin has been extensively studied in animal reproduction, the mechanism of melatonin in puberty remains elusive. This study was designed to explore the effect of intraperitoneal administration of melatonin on puberty onset in female mice. The injection of melatonin into postnatal days 10 mice at a dose of 15 mg/kg accelerated the puberty onset in mice. Mechanistically, there was no difference in physical growth and serum Leptin levels after melatonin administration. Meanwhile, the serum levels of reproductive hormones involved in hypothalamic-pituitary-ovarian axis, such as FSH and estrogen level in serum were increased. The mRNA levels of *GnRH* and *GnRHr* were not affected by melatonin, while the expressions of *FSHβ* in pituitary and *Cyp19a1* in ovary were significantly up-regulated. In addition, melatonin still promoted FSH synthesis after ovariectomy. Furthermore, the enhanced activity of ERK1/2 signaling verified that the expression of *FSHβ* increased in pituitary. We confirmed that melatonin promoted the FSH synthesis in pituitary, thereby increased serum estrogen levels and ultimately accelerated puberty onset. However, these effects of melatonin may be pharmacological due to the high dose. This study would help us to understand the functions of melatonin in pubertal regulation comprehensively.

## 1. Introduction

Melatonin (MLT) is synthesized mainly in the pineal gland related to the control of the circadian rhythm [1] and seasonal reproduction [2]. In addition, melatonin is a well-known effective antioxidant which served as a scavenger of free radicals or a metabolite playing a cascade amplification of antioxidant effects in cells [3]. In humans, melatonin has been widely used in the fields of anti-aging, anti-cancer, and hypnosis [4,5,6]. In female animals, melatonin plays an important role in reproductive hormone secretion, oocyte quality, luteal function, ovulation, and early embryonic development [7,8]. Further studies showed that melatonin regulates the secretion of FSH and LH mainly through the hypothalamus-pituitary-gonadal axis and then affect the secretion of gonadal reproductive hormones, or directly acts on ovarian melatonin receptors to regulate the secretion of steroid sex hormones [9,10]. There are two melatonin receptors in mammals, melatonin receptor 1 (MT1) and melatonin receptor 2 (MT2) [11,12]. It was recently demonstrated that melatonin is synthetized in brain mitochondria and acts through the mitochondrial external membrane MT1 in mice [13]. Secretion of luteinizing hormone (LH) before ovulation can result in high expression of *MTI* in ovarian granulosa cells. Meanwhile, the expression level of melatonin synthase *SNAT* (*serotonin N-acetyltransferase*) in cumulus cells increases significantly, and the level of melatonin in follicular fluid also increases [14]. The level of progesterone in mouse follicles significantly increases when treated with melatonin in vitro [15]. The addition of exogenous melatonin significantly mitigated the oxidative damage of matured bovine oocytes [16], and prominently improved the developmental potential of fertilized embryos in vitro [17].

Female puberty means the first appearance of estrus and ovulation, which is a sign of obtaining fertility. The length of the puberty period is reliably correlated with the animal’s fecundity, and the puberty is regulated by the hypothalamic-pituitary-ovarian axis. It has become clear that an increased secretion of the gonadotropin-releasing hormone (*GnRH*) from the hypothalamus triggers the onset of puberty [18]. Puberty is initiated by the emergence of pulsatile *GnRH* secretion at a reproductively appropriate time. The basal level of melatonin in the blood declines significantly with the release of pituitary gonadotropin, which suggests that melatonin may be involved in the regulation of animals’ puberty [19]. A previous study has shown a decrease of plasma melatonin at the onset of puberty with the growth of age, the decrease of serum melatonin may contribute to the activation of hypothalamic pulsatile *GnRH*, which in turn activates the reproductive axis and promotes the occurrence of puberty [20]. Nutritional conditions and the amount of body energy reserves have been attested to the critical role in regulating the onset of puberty [21], and Leptin is an important basis for the link between animal nutrition and reproductive function [22]. Studies found that *Leptin* mRNA levels, steroid hormone secretion and litter size reduces in MT1 knockout mice [23].

The effect of melatonin on puberty has been studied extensively in recent years. Melatonin has no effect on development of adolescents who received long-term melatonin treatment [24]. Studies in gilts indicated that increase of serum melatonin via implants did not alter the onset of puberty [25]. Studies of ewe lambs found that melatonin can overcome the endocrine and metabolic disorders of the puberty, then stimulate the onset of puberty, and improve reproductive performances [26,27]. However, melatonin showed a negative function in male hamsters, which delayed the onset of puberty [28]. To sum up, melatonin has different influences on puberty in various juvenile animals. Nevertheless, the function of melatonin on puberty onset remains to be determined in mice. This study was performed to verify whether exogenous melatonin can regulate the puberty onset in female mice, and the results showed that exogenous melatonin accelerated the puberty onset.

## 2. Results

### 2.1. Melatonin Administration during Juvenile Stage Accelerated the Onset of Puberty

**Figure 1:**
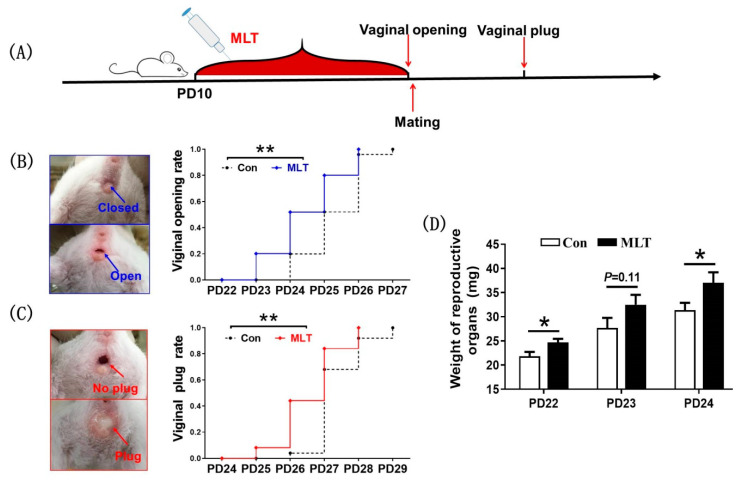
Melatonin administration during juvenile stage accelerated the onset of puberty. (**A**) The diagram of experimental design. (**B**) The effect of melatonin on vaginal opening: the representative photographs of vaginal opening and the statistic chart of vaginal opening. Con: *n* = 25; MLT: *n* = 25. df = 48; t = 3.113. (**C**) The effect of melatonin on mating date: the representative photographs of mating plug and the statistic chart of mating date. Con: *n* = 25; MLT: *n* = 25. df = 48; t = 3.246. (**D**) The effect of melatonin on reproductive weight. PD22 (Con: *n* = 19; MLT: *n* = 19. df = 36; t = 2.413.), PD23 (Con: *n* = 18; MLT: *n* = 17. df = 33; t = 1.627.), PD24 (Con: *n* = 20; MLT: *n* = 19. df = 37; t = 2.176.). Data were showed as “mean ± SEM”. * represent significant differences, *p* < 0.05; ** represent significant differences, *p* < 0.01.

To investigate the role of melatonin on juvenile mice, estrus performance was observed after injection of melatonin. The results indicated that the average day of puberty onset in melatonin-treated group was significantly earlier than that in control group (24.48 ± 0.21 vs 25.32 ± 0.17, *p* < 0.05), respectively (Figure 1B). Meanwhile, the average mating date in melatonin-treated group was also significantly earlier than that in control group (26.64 ± 0.17 vs 27.36 ± 0.14, *p* < 0.05), respectively (Figure 1C). Subsequently, we dissected and weighed the reproductive organs, and the results displayed that melatonin significantly increased the weight of the reproductive organs at PD22 (24.7 ± 0.76 vs 21.8 ± 0.91, mg, *p* < 0.05) and PD24 (37.0 ± 2.15 vs 31.4 ± 1.52, mg, *p* < 0.05), respectively (Figure 1D).

### 2.2. Melatonin Did Not Accelerate the Onset of Puberty by Physical Development and Leptin Level

**Figure 2:**
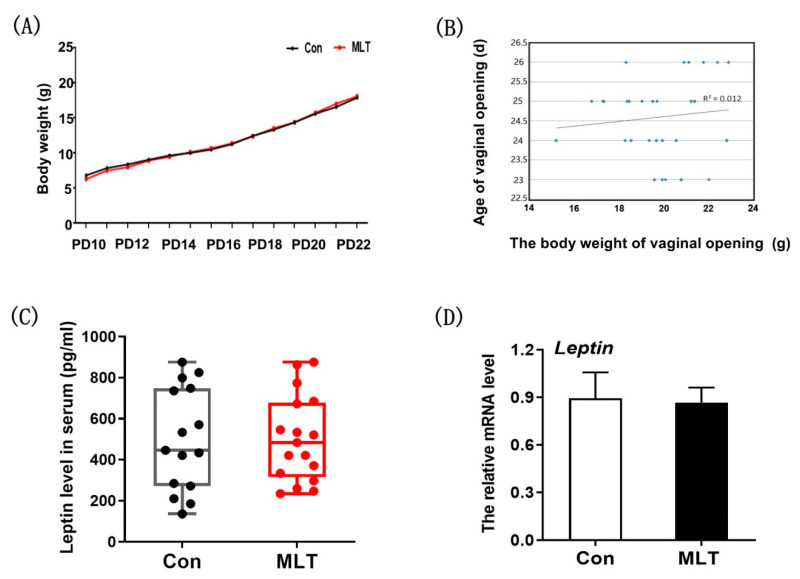
Melatonin did not accelerate the onset of puberty by physical development and Leptin level. (**A**) The effect of melatonin on growth curve of mice. Con: *n* = 4; MLT: *n* = 4. (**B**) The correlation analysis between body weight and vaginal opening. *n* = 30. (**C**) The effect of melatonin on Leptin level in serum. Con: *n* = 15; MLT: *n* = 17. df = 30; t = 0.047. (**D**) The effect of melatonin on Leptin level in adipose tissue. Con: *n* = 10; MLT: *n* = 10. df = 18; t = 0.446. qRT-PCR was independently repeated at least three times. Data were showed as “mean ± SEM”.

There is a close relationship between reproductive performance and nutrient level in mammalian. Therefore, we speculated that melatonin affected the onset of puberty by affecting physical development and Leptin level. It was observed that melatonin did not affect the growth curve of mice (Figure 2A). Meanwhile, there was no significant correlation between body weight and vaginal opening time (R^2^ = 0.012) (Figure 2B). The expression level of Leptin in serum and adipose tissue showed no significant differences after melatonin administration (Figure 2C,D).

### 2.3. Melatonin Accelerated the Onset of Puberty by Enhancing the Secretion of FSH and Estrogen

**Figure 3:**
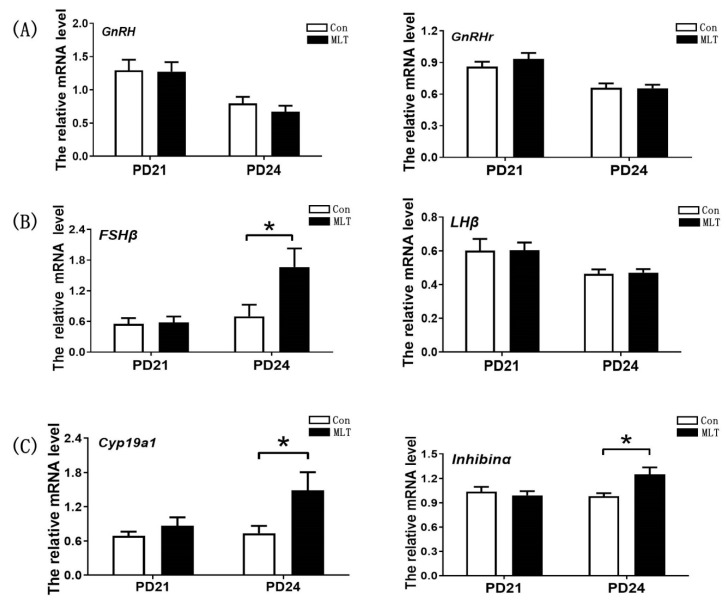
Melatonin accelerated the onset of puberty by enhancing the secretion of FSH and estrogen. (**A**–**C**) The effect of melatonin on the expression of genes in hypothalamus-pituitary-ovarian axis. Con: *n* = 7; MLT: *n* = 7. *Fshβ*: df = 12; t = 2.249(PD24). *Cyp19a1*: df = 12; t = 2.201(PD24). *Inhibinα*: df = 12; t = 2.827(PD24). The sample size of hormones assay was 21 and 24 in each group; qRT-PCR was independently repeated at least three times. Data were showed as “mean ± SEM”. * represent significant differences, *p* < 0.05.

To further elucidate the potential mechanism of melatonin in regulating puberty, we examined the secretion of hormones and expression levels of genes involved in the hypothalamic-pituitary-ovarian axis. It was observed that melatonin significantly increased FSH level at PD23 (1.64 ± 0.118 vs 1.22 ± 0.138, mIU/mL, *p* < 0.05) and PD24 (1.60 ± 0.096 vs 1.26 ± 0.130, mIU/mL, *p* < 0.05) respectively. Estrogen was also up-regulated by melatonin at PD21 (18.08 ± 1.307 vs 11.96 ± 1.641, pg/mL, *p* < 0.01), PD22 (22.39 ± 2.128 vs 14.04 ± 1.109, pg/mL, *p* < 0.01) and PD24 (13.97 ± 1.074 vs 10.82 ± 1.103, pg/mL, *p* < 0.05) respectively. However, the level of LH did not significantly changed after melatonin administration (Table 1). *GnRH* and *GnRHr* were not affected by melatonin (Figure 3A), suggesting that the positive impact of melatonin on FSH synthesis was not mediated by hypothalamic. Consistent with FSH level in serum, the level of *FSHβ* in anterior pituitary presented the significantly increase by melatonin at PD24 (1.66 ± 0.363 vs 0.70 ± 0.228, *p* < 0.05), while the expression level of *Lhβ* was not affected by melatonin (Figure 3B). In the ovary, the expression level of *Cyp19a1*, was significantly increased by melatonin at PD24 (1.49 ± 0.317 vs 0.74 ± 0.130, *p* <0.05), and *Inhibinα* was also up-regulated by melatonin at PD24 (1.25 ± 0.087 vs 0.98 ± 0.040, *p* <0.05) (Figure 3C). These data suggested that melatonin may promote the puberty onset in juvenile mice by affecting pituitary or ovary.

### 2.4. Melatonin Accelerated the Onset of Puberty by the Pituitary

**Figure 4:**
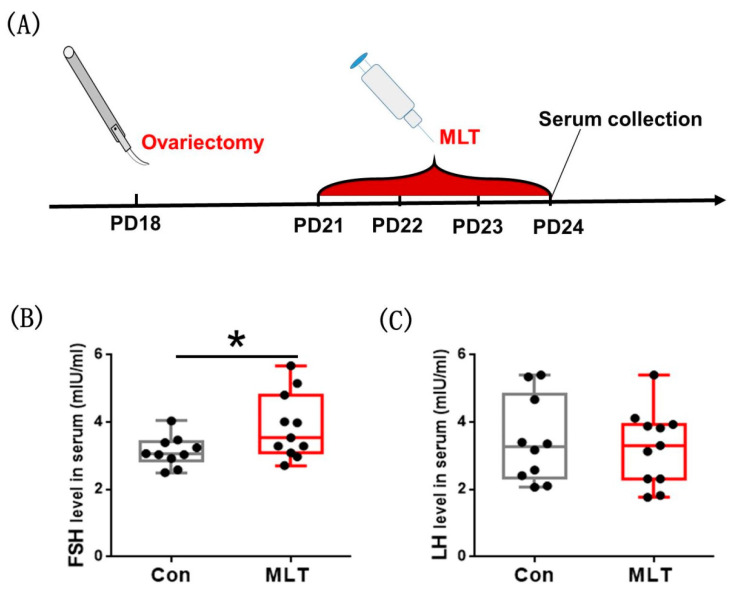
Melatonin accelerated the onset of puberty by the pituitary. (**A**) The diagram of experimental design. (**B**) The effect of melatonin on FSH level in serum after ovariectomy. Con: *n* = 10; MLT: *n* = 11. df = 19; t = 2.197. (**C**) The effect of melatonin on LH level in serum after ovariectomy. Con: *n* = 10; MLT: *n* = 11. df = 19; t = 0.570. Data were showed as “mean ± SEM”. * represent significant differences, *p* < 0.05.

To verify whether melatonin promoting puberty onset is ovarian-mediated, we removed mice ovaries at PD18, performed melatonin injection subsequently during PD21–24, and finally collected the serum for assay (Figure 4A). It was observed that FSH level in serum was significantly elevated by melatonin (3.865 ± 0.292 vs 3.130 ± 0.141, mIU/mL, *p* < 0.05) (Figure 4B). The level of LH was not affected by melatonin injection (Figure 4C). Melatonin still promoted FSH synthesis after ovariectomy, which demonstrated that melatonin accelerated the onset of puberty by the pituitary.

### 2.5. Melatonin Promoted the FSH Synthesis through the Pituitary ERK1/2 Signaling Pathway

**Figure 5:**
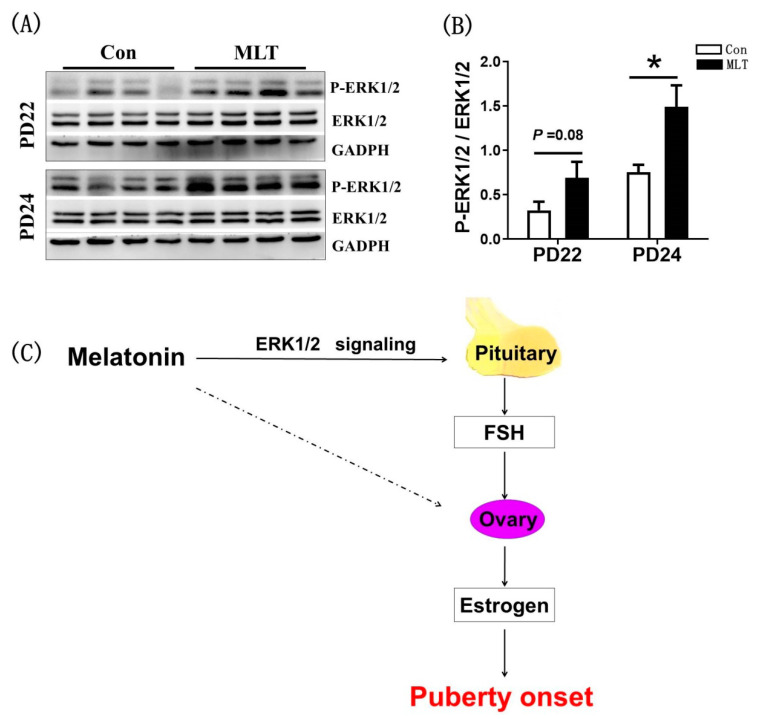
Melatonin promoted the FSH synthesis through the pituitary ERK1/2 signaling pathway. (**A**) The effect of melatonin on the phosphorylation of ERK1/2. (**B**) The statistic of P-ERK1/2/ERK1/2. Con: *n* = 4; MLT: *n* = 4. df = 6; t = 2.87. (**C**) The pattern diagram of melatonin mediating puberty onset. Western blot was independently repeated 4 times. Data were showed as “mean ± SEM”. * represent significant differences, *p* < 0.05.

To verify the effect of melatonin on the onset of puberty is mediated by the pituitary, we detected the activity of ERK1/2 pathway that controls the expression of *FSHβ* in anterior pituitary by western blot (Figure 5A). It was observed that melatonin significantly enhanced the phosphorylation level of ERK1/2 at PD24 (1.49 ± 0.244 vs 0.75 ± 0.083, *p* < 0.05)) (Figure 5B). We confirmed that melatonin promoted the FSH synthesis by enhanced activity of ERK1/2 signaling.

## 3. Discussion

This study indicated that melatonin accelerated the onset of puberty in female mice, and then simultaneously identified the mediation pathway. Other studies showed that body weight is the primary factor controlling puberty onset [29,30]. The puberty can only be induced when the females develop to a certain body weight. Therefore, we firstly examined the effect of melatonin on mice growth. The results showed that melatonin treatment had no effect on growth (Figure 2A), which is consistent with results from previous studies comparing melatonin effect on domestic cats’ body weight [31]. Meanwhile, there was no significant correlation between body weight and puberty onset (Figure 2B). It has been shown that Leptin can promote the onset of puberty and the maturation of the hypothalamic-pituitary-gonadal axis in mammals, especially stimulate *GnRH* secretion before puberty [32], which may affect the occurrence of puberty [33]. Therefore, we conjectured melatonin may affected the puberty onset by regulating the expression of *Leptin*, however, the results showed that there was no significant difference between the two groups. Further study should be instituted into the underlying mechanism of melatonin in regulating puberty.

The regulation of puberty was related to the body growth and the hypothalamic-pituitary-ovarian axis, we examined the secretion of hormones and expression of genes involved in the hypothalamic-pituitary-ovarian axis, and the result showed that the serum levels of FSH and estrogen were significantly increased (Table 1), which may directly accelerated the puberty onset. In the hypothalamic-pituitary-gonadal axis, FSH and estrogen are directly upstream and downstream relationship, forming a mutual feedback regulatory loop. According to the results, it could be conjectured that the positive impact of melatonin on FSH synthesis was not mediated by hypothalamic, as the mRNA levels of *GnRH* and *GnRHr*, the crucial genes in *GnRH* signaling that controls FSH synthesis [34], were not affected by melatonin (Figure 3A). Meanwhile, the expressions of FSH synthesis gene *FSHβ* [35] and regulatory gene *Inhibinα* [36] were significantly increased by melatonin. The expression of *Cyp19a1*, which is related to the synthesis of estrogen [37], was also significantly enhanced. These data suggested that melatonin promoted the puberty onset in juvenile mice through the pituitary or directly on the ovary. Ovariectomy was performed to verify whether ovary mediates the impact of melatonin on puberty onset. Notably, melatonin still promoted FSH synthesis in ovariectomized mice (Figure 4B), which demonstrated that melatonin accelerated the puberty onset not by ovary. Moreover, we observed that melatonin significantly enhanced the level of phosphorylated ERK1/2, a pivotal signaling pathway that controls the transcription of *FSHβ* in the pituitary [38]. These findings in the present study are not consistent with those in a previous study where it was reported that the most definitive endocrine characteristic of puberty is the change of *GnRH* release. Promotion of the release of FSH and LH, the gradual elevation of these hormones, particularly LH, over the pubertal period allows for ovarian development and subsequent secretion of estrogen [39,40].

In the present study, the dose of 15 mg/kg melatonin was effective in accelerating puberty onset, we should also be aware that the above conclusion was obtained by treating mice with high-dose melatonin. Whether the physiological level of melatonin has the ability to regulate puberty remains to be elucidated. Although these results of the current study indicated that melatonin accelerated puberty onset by promoting the FSH synthesis in pituitary, the specific pathway of this function is still unknown. The issues whether melatonin effects pituitary directly or by other substances remain to be further investigated. This study implied that melatonin could be useful for advancing reproductive capacity in mice, as we observed that melatonin treated pubertal mice can be mated normally (Figure 1C). In the future, how does melatonin regulate pituitary function will be the focus of our work.

## 4. Materials and Methods

### 4.1. Ethics

KM-strain mice were purchased from the Center for Animal Testing of Huazhong Agricultural University. Mice were housed in a temperature controlled facility (24 ± 2 °C) with constant 12 h light–dark cycles. Animals were allowed to access to food and water ad libitum. Animal feeding and tests were conducted based on the National Research Council Guide for the Care and Use of Laboratory Animals and approved by the Institutional Animal Care and Use Committee. All experimental protocols involving animal handling in this study were conducted in accordance with the requirements of the Institutional Animal Ethics Committee of Huazhong Agricultural University (approval number: HZAUMO−2018−058).

### 4.2. Experimental Design and Animal Treatment

The juvenile mice at postnatal days 10–24 were chosen for this research. Mice received melatonin (Sigma, St. Louis, MO, USA) intraperitoneally every morning (8:00 AM) at doses of 15 mg/kg (melatonin was dissolved in alcohol and diluted to the required dose with sterile saline to a final concentration of 5%). The dosage of melatonin was 15 mg/kg based on previous literatures [41,42,43,44].

To determine the effect of melatonin on puberty onset, melatonin was injected into mice every morning from day 10 to the day of vaginal opening, 25 mice in each group were used in this experiment. The day of vaginal opening was recorded as puberty onset, and the day vaginal plug appeared was recorded as the first mating age. The reproductive organs at days 22, 23, and 24 were collected and weighed, 17–20 mice in each group were used in this experiment. To examine the mechanism of melatonin on the onset of puberty, the growth curve of mice in each group was measured; the blood samples and adipose tissue at days 24 were collected to detect serum Leptin levels, 15–17 mice in each group were used in this experiment; the blood samples at days 21–24 were collected to measure hormone levels (FSH, LH and estrogen), 17–21 mice in each group were used in this experiment. The pituitary, hypothalamus and ovaries were collected at days 21 and 24 for qRT-PCR, seven mice in each group were used in this experiment. Meanwhile, ovariectomy was conducted at postnatal days 18, and then melatonin was injected in mice during postnatal days 21–24 to verify whether ovary affects melatonin on puberty onset, 10–11 mice in each group were used in this experiment. Pituitary was collected at days 22 and 24 for western blot assays, four mice in each group were used in this experiment. Euthanasia was performed using cervical dislocation after ether anesthesia. All mice were sacrificed at the end of the experiment.

### 4.3. Hormone Determination

For reproductive hormones detection, blood samples of each group were collected via the eyelid venous under anesthesia. After clotting for 30 min, the serum was obtained by centrifugation at 3000 rpm for 10 min and stored at −20 °C. The serum levels of hormones were detected by radioimmunoassay. The detection work was entrusted to Beijing North Institute of Biological Technology (Beijing, China). The information of detection kits is as follows: FSH, #B03PZB, Beijing North Institute of Biological Technology, Beijing, China; LH, #B04PZB, Beijing North Institute of Biological Technology, Beijing, China; estrogen, #B05PZB, Beijing North Institute of Biological Technology, Beijing, China.

### 4.4. Real-Time Quantitative PCR Analysis

Total RNA was extracted using the Trizol reagent (Invitrogen Inc., Carlsbad, CA, USA). Qualified RNA samples (260/280 & 260/230 ≥ 2) were used for reverse transcription. The PrimeScriptTM RT reagent Kit with genome DNA Eraser (Takara Bio Inc., Dalian, China) was used to obtain reverse transcription. Real-time qPCRs were run using a Bio-rad CFX Manager Machine (Bio-Rad, Hercules, CA, USA). The real-time qPCR system consisted of SYBR Green (5 µL) from a QuantiFast SYBR Green PCR Kit, forward and reverse primers (30 µM, 1 µL), template cDNA (4 µL) was added up to a total volume of 10 µL. The procedure was as follows: 95 °C for 10 min; 35 cycles of 95 °C for 10 s and 60 °C for 15s and a melting curve from 65 °C to 95 °C with the ramp rate 0.1 °C/s. A melting curve analysis was subsequently performed to confirm that a single specific product was generated. The housekeeping gene *Actb* was used as a control for calibration. Relative mRNA expression was calculated by the 2^−^^△△ct^ method. Primer sequences are listed in Table 2.

### 4.5. Western Blotting

Total protein was extracted from the pituitary tissue with RIPA lysis buffer (Servicebiotech technology, Wuhan, China), with addition of 1 mM of phosphorylase inhibitor and phenylmethanesulphonyl fluoride (PMSF). The proteins were heated at 100 °C for 5 min, then separated via sodium dodecyl sulphate-polyacrylamide gel electrophoresis (SDS–PAGE) with a 5% stacking gel and a 10% separating gel and were subsequently transferred to polyvinylidene fluoride (PVDF) membranes (Servicebiotech technology, Wuhani, China) via electrophoresis. The membranes were first incubated for 3 h in 5% skim milk in Tris-buffered saline containing Tween (TBST; Tris–HCl containing 0.1% Tween 20) and subsequently incubated overnight at 4 °C with primary antibodies for the following proteins: ERK (#GB11560; 1:1500, Servicebiotech technology, Wuhani, China), phosphor-ERK (#AP0472; 1:1000, Abclonal, Wuhan, China), GAPDH (#CW0100M; 1:2000; CWBiotech, Beijing, China). Afterward, the membranes were washed with TBST and incubated with the corresponding secondary antibody (goat anti rabbit IgG: #BF03008, 1:4000, Biodragon-immunotech, Beijing, China; goat anti mouse IgG: #CW0102S, 1:4000, CWBiotech, Beijing, China) at room temperature for 2 hrs. Finally, the membranes were washed in TBST, and the immunoblots were visualized with an ECL kit (CWBiotech, Beijing, China) and developed using a chemiluminescence system (Thermo Scientific, Waltham, MA, USA).

### 4.6. Statistics Analysis

Data are expressed as the mean ± S.E.M. Statistical analyses were performed using the unpaired Student’s t-test with GraphPad Prism 6.01. *p* < 0.05 was considered to be statistically significant, and *p* < 0.01 was considered to be highly statistically significant.

## 5. Conclusions

In conclusion, intraperitoneal administration of melatonin to juvenile female mice accelerated the onset of puberty. The mechanistic studies indicated that melatonin application promoted the FSH synthesis by activating ERK1/2 signaling in pituitary, thereby increased estrogen levels and ultimately accelerated puberty onset (Figure 5C). This finding provides a potential function of melatonin in animal puberty.

## Figures and Tables

**Table 1:** The effect of melatonin on FSH, LH and estrogen levels in serum.

Hormones	Postnatal Days	Con	MLT	df/t
FSH mIU/mL	PD21	1.37 ± 0.232 ^a^	1.33 ± 0.171 ^a^	38/0.151
	PD22	0.93 ± 0.156 ^a^	1.07 ± 0.170 ^a^	36/0.592
	PD23	1.22 ± 0.138 ^a^	1.64 ± 0.118 ^b^	37/2.295
	PD24	1.26 ± 0.130 ^a^	1.60 ± 0.096 ^b^	38/2.158
LH mIU/mL	PD21	4.82 ± 0.643 ^a^	4.17 ± 0.559 ^a^	36/0.768
	PD22	5.41 ± 0.682 ^a^	5.30 ± 0.496 ^a^	37/0.121
	PD23	4.84 ± 0.455 ^a^	5.20 ± 0.592 ^a^	35/0.489
	PD24	4.11 ± 0.598 ^a^	3.32 ± 0.450 ^a^	38/1.051
Estrogen pg/mL	PD21	11.96 ± 1.641 ^a^	18.08 ± 1.307 ^b^	34/2.942
	PD22	14.04 ± 1.109 ^a^	22.39 ± 2.128 ^b^	37/3.340
	PD23	14.82 ± 1.959 ^a^	20.51 ± 2.370 ^a^	35/1.807
	PD24	10.82 ± 1.103 ^a^	13.97 ± 1.074 ^b^	36/2.040

Note: In each group *n* = 17–21. The different superscript letters (a, b) represent significant differences (*p* < 0.05).

**Table 2:** PCR primers used for SYBR green Q-PCR analysis.

Genes	Primer seq (5′–3′)	Size (bp)
*Actb*	F: CCAGCCTTCCTTCTTGGGTAT R: AGGTCTTTACGGATGTCAACG	93
*Leptin*	F: CAAGCAGTGCCTATCCAGAAA	162
	R: GGACAAACTCAGAATGGGGTG	
*GnRH*	F: AGGAAGCCAGGCAGAAGAAG	100
	R: GAGCCATTAACAGGTCACAAGC	
*GnRH* *r*	F: GATGGTGGTGATTAGCCTGGAC	192
	R:CATTGCGAGAAGACTGTGGG	
*FSHβ*	F: ACCACTCATCCCTCCATCCA	166
	R: CCACTTTCCTTTCCTCCCTCTA	
*LHβ*	F: CCCATAGTCTCCTTTCCTGTAGC	97
	R: AGGCCATTGGTTGAGTCCTG	
*Cyp19a1*	F: GACACATCATGCTGGACACC	179
	R: CAAGTCCTTGACGGATCGTT	
*Inhibinα*	F: CTTTCCCTCTGCTGACCCA	184
	R: AAAGCCGCAGGAGACCAA	

## Data Availability

The data presented in this study are available on request from the corresponding author.

## Sample Availability

Samples of the compounds are not available from the authors.
